# P41 Decoding short stature in a child with lupus nephritis: an unusual association with de la Chapelle syndrome

**DOI:** 10.1093/rap/rkae117.072

**Published:** 2024-11-05

**Authors:** Prabal Barman, Reva Tyagi, Roshan Daniel, Ankur Kumar Jindal, Amit Rawat

**Affiliations:** Paediatric Allergy Immunology Unit, Department of Paediatrics, Advanced Paediatrics Centre, PGIMER, Chandigarh, India; Paediatric Allergy Immunology Unit, Department of Paediatrics, Advanced Paediatrics Centre, PGIMER, Chandigarh, India; Genetic and Metabolic Unit, Department of Paediatrics, Advanced Paediatrics Centre, PGIMER, Chandigarh, India; Paediatric Allergy Immunology Unit, Department of Paediatrics, Advanced Paediatrics Centre, PGIMER, Chandigarh, India; Paediatric Allergy Immunology Unit, Department of Paediatrics, Advanced Paediatrics Centre, PGIMER, Chandigarh, India

## Abstract

**Introduction:**

Recent studies have implicated the dysregulation of epigenetic factors in maintaining favourable lyonization of X-chromosome as a plausible putative mechanism in causing high incidence of systemic lupus erythematosus (SLE) in females. This is reflected by the increased risk of SLE in patients with ‘disorders of sex development’ (DSD) such as Klinefelter’s syndrome (47,XXY). de la Chapelle syndrome (46,XX with *SRY* gene) is another DSD wherein patients resemble males phenotypically with 2 X-chromosomes in their karyotype. Herein, we report the diagnostic and therapeutic challenges in a case of juvenile-onset SLE with refractory lupus nephritis and concomitant association of de la Chapelle syndrome.

**Case description:**

A 13-year-old boy presented with history of chronic febrile inflammatory polyarthritis involving small and large joints for 6-months. On examination, he had short stature, and swelling, tenderness and restriction of movement of the proximal and distal interphalangeal joints, wrist and shoulder joints of both sides. Investigations revealed elevated erythrocyte sedimentation rate with normal C-reactive protein. Routine urine microscopy examination was suggestive of proteinuria. Further immunological investigations revealed hypocomplementemia, positive anti-nuclear antibody (4+, homogeneous pattern) and elevated titres of anti-double stranded DNA. He was also noted to have positive direct coomb’s test and antiphospholipid antibodies. His renal biopsy was suggestive of lupus nephritis (Class II). Based on the above constellation of clinical and laboratory parameters, a diagnosis of SLE with lupus nephritis (Class II) and musculoskeletal involvement was proffered. He was started on oral prednisolone (2 mg/kg/day followed by taper), hydroxychloroquine (5 mg/kg/day) and sun protective measures. However, persistence of proteinuria after 3-months necessitated addition of mycophenolate mofetil (1200 mg/m^2^/day) following which his SLE-disease activity went into remission.

Notwithstanding the control of SLE-disease activity, there was no improvement in his height after 1-year of follow-up. A pediatric endocrinology consultation for short stature revealed features of idiopathic growth hormone (GH) deficiency and primary hypogonadism. Chromosomal analysis was suggestive of 46,XX karyotype, and fluorescence in situ hybridisation revealed ‘XX’ pattern with *SRY* gene located on one of the X chromosomes. He was commenced on recombinant GH therapy for 18-months following which his height improved.

At 2-years of follow-up, he developed worsening proteinuria. A repeat renal biopsy was suggestive of class-IV lupus nephritis. However, he responded poorly to 6-doses of monthly intravenous cyclophosphamide (500 mg/m^2^/dose). Considering refractory lupus nephritis, he was commenced on rituximab [(375 mg/m^2^/dose) at 0 and 2-weeks] following which his proteinuria became passive. He remains well on follow-up.

**Discussion:**

SLE in DSD has been more frequently described in the context of Klinefelter’s syndrome (47, XXY), implicating the X chromosome as a putative mechanism in its pathogenesis. However, the clinical profile and outcome of SLE in patients with de la Chapelle syndrome has not been explored extensively. de la Chapelle syndrome (46, XX with *SRY* gene), first described in 1964, is a rare DSD with a reported prevalence of 1/20000 to 1/25000 newborn males. The clinical phenotype includes decreased body height, gynecomastia, maldescended testis and other features of hypogonadism including infertility. Chagnon et al reported the first patient of SLE in de la Chapelle syndrome. Authors described a 19-year-old man who presented with features of SLE since early childhood. His disease was complicated by severe rash, mucositis, arthritis, focal proliferative glomerulonephritis, transaminitis and refractory thrombotic thrombocytopenic purpura, and osteoporosis (including growth failure). In addition, he also had primary hypogonadism with normal GH. Dillon et al also reported another 46, XX male with presence of *SRY* gene. However, the clinical details including treatment were not elucidated in this paper. Both these patients were phenotypically male due to the presence of *SRY* gene, similar to the index patient. Our patient’s difficult clinical course with arthritis, growth failure and refractory lupus nephritis, and primary hypogonadism was similar to the patient reported by Chagnon et al. The treatment protocol of SLE in DSD is not well-established. Chagnon et al reported using corticosteroids, hydroxychloroquine, methotrexate, intravenous pulse cyclophosphamide, azathioprine, and plasmapheresis. The index patient was treated similarly; however, due to refractory lupus nephritis, he was also given rituximab. To the best of our knowledge, this is the first report of successful use of anti-CD20 therapy in SLE with DSD such as de la Chapelle syndrome.

**Key learning points:**

• This case highlights the challenges in diagnosis and management of patients with DSD including de la Chapelle syndrome. The main learning point from this case was that short stature in juvenile-onset SLE in a boy with features of primary hypogonadism warrants further evaluation. As reported in previous literature, this subset of patients may not respond adequately to conventional first-line therapy. The need for escalation of therapy or primary therapy with rituximab in such patients needs further exploration. Another point of concern is using cyclophosphamide, a known drug to have adverse effect on testes, in these patients since it may further compromise testicular function.

